# Hidden in the Mediastinum: A Grade 1 Neuroendocrine Tumor Revealed by Refractory Hypokalemia and Ectopic Adrenocorticotropic Hormone (ACTH)-Dependent Cushing's Syndrome

**DOI:** 10.7759/cureus.107490

**Published:** 2026-04-21

**Authors:** Sofía Paola Agüero-Pineda, Rodrigo Grimaldo-Rivera, José Abraham Camacho-Muñoz, Ana Theresa Martínez-Navarro, Ernesto Landeros-Navarro, Sol Ramírez-Ochoa, Miguel Ángel Zúñiga-García, Diego A. Zúñiga-Tamayo, Mónica Rubio-Martín del Campo, Berenice Vicente-Hernández, Enrique Cervantes-Pérez

**Affiliations:** 1 Department of Internal Medicine, Hospital Civil de Guadalajara Fray Antonio Alcalde, Guadalajara, MEX; 2 Department of Pathology, Hospital Civil de Guadalajara Fray Antonio Alcalde, Guadalajara, MEX; 3 School of Medicine, Centro Universitario de Tlajomulco, University of Guadalajara, Tlajomulco de Zúñiga, MEX; 4 Department of Medicine, Health Sciences University Center, University of Guadalajara, Guadalajara, MEX

**Keywords:** cushing's syndrome, ectopic acth syndrome (eas), hypercortisolism, hypokalemia, video-assisted thoracoscopic surgery (vats)

## Abstract

Ectopic adrenocorticotropic hormone (ACTH)-dependent Cushing's syndrome (CS) is a rare, fulminant variant of endogenous hypercortisolism that demands rapid localization of the ACTH source to achieve a cure. A 51-year-old woman presented with progressive proximal muscle weakness, centripetal obesity, new-onset hypertension with a blood pressure of 150/90 mmHg at arrival, and persistent hypokalemia (2.2 mmol/L). Biochemical testing confirmed severe hypercortisolism that failed to suppress after an overnight 1-mg dexamethasone test. Plasma ACTH was markedly elevated (798 pg/mL), indicating ACTH-dependent disease. Pituitary MRI showed no adenoma, so an ectopic source was suspected. Contrast-enhanced thoracoabdominal CT revealed a 24 × 14-mm anterior mediastinal node, which was excised via video-assisted thoracoscopic surgery (VATS). Histopathology confirmed a grade 1 neuroendocrine tumor with diffuse positivity for synaptophysin, chromogranin A, and neuron-specific enolase. Twenty-four hours after surgery, serum cortisol decreased to 11.1 µg/dL and ACTH to 2.0 pg/mL; serum potassium normalized without supplementation. Octreotide receptor scintigraphy showed no residual or metastatic disease, and the patient remained normotensive and normokalemic at follow-up. Rapidly progressive hypercortisolism accompanied by refractory hypokalemia should prompt consideration of ectopic ACTH secretion. A stepwise diagnostic approach, including biochemical confirmation, exclusion of pituitary disease, and targeted thoracic localization, enabled early minimally invasive resection and biochemical cure in this case. Early recognition and surgery remain the cornerstones of management for ectopic ACTH-producing neuroendocrine tumors. Prompt identification and anatomical localization of the ectopic source are essential to prevent prolonged exposure to severe hypercortisolism and its associated metabolic and cardiovascular complications. Timely surgical resection allows rapid biochemical remission and improvement in short- and long-term outcomes.

## Introduction

Cushing's syndrome (CS) is a chronic multisystem disorder most commonly caused by prolonged exposure to exogenous glucocorticoids. Diagnosis becomes more challenging when the etiology is endogenous. Endogenous CS is classified into two major etiologic groups: adrenocorticotropic hormone (ACTH)-dependent disease, accounting for 70% to 80% of cases, and ACTH-independent disease. The most frequent ACTH-dependent cause is a corticotroph pituitary adenoma (Cushing's disease), present in 80% to 90% of cases, whereas ectopic CS (ECS) represents the remaining cases [1]. In ECS, ectopic ACTH secretion arises predominantly from pulmonary neuroendocrine neoplasms (NENs), mainly pulmonary carcinoid tumors and small-cell lung cancers, accounting for 25% and 20% of cases, respectively. The clinical manifestations of ECS depend on the severity of hypercortisolism, which in turn depends on the duration and production rate of ectopic corticosteroid. Patients typically present with abrupt weight gain, proximal muscle weakness, hypokalemic metabolic alkalosis, and markedly elevated serum cortisol levels, contrasting with the more indolent course of pituitary ACTH-dependent CS [2]. ECS is most commonly caused by NENs located in the thorax or upper abdomen; bronchial NENs account for 20% to 40% of cases, and other intrathoracic tumors for 50% to 80% of reported cases [3]. Initial screening should include at least two of the following tests: late-night salivary cortisol, 24-hour urinary free cortisol, or the overnight 1-mg dexamethasone suppression test. Hypercortisolism is confirmed when at least two tests are positive. To differentiate pituitary from ectopic ACTH production, three noninvasive studies are recommended: pituitary MRI, the high-dose dexamethasone suppression test, and the corticotropin-releasing hormone (CRH) stimulation test. In confirmed ECS, contrast-enhanced CT (CECT) from the neck to the pelvis is recommended to localize tumors; CT is superior to MRI for thoracic lesions. Functional imaging is valuable for detecting hidden tumors, confirming the neuroendocrine nature of lesions, and staging malignancy [3]. Gallium-68 somatostatin receptor positron-emission tomography/CT (68Ga-SSTR PET/CT) identified tumors in 52% of ECS cases. Indium-111 pentetreotide scintigraphy historically detected approximately half of ECS tumors, but its use has declined due to the superior sensitivity of the newest imaging modalities [4]. Definitive management of ECS consists of complete surgical resection of the tumor; however, this is not always feasible in metastatic or occult disease. In such cases, hypercortisolism may be controlled with steroidogenesis inhibitors, glucocorticoid receptor antagonists, and/or adrenolytic agents. Radiotherapy is reserved for persistent primary lesions, incomplete resections, inoperable tumours, or bronchial and thymic neoplasms [3].

ECS represents a considerable diagnostic and therapeutic challenge due to its insidious clinical presentation and the severity of symptoms associated with hypercortisolism, and requires a structured workup to identify the origin of the endogenous production of cortisol. In this context, we present a case report of a patient with clinical and biochemical manifestations suggestive of ACTH-dependent hypercortisolism, whose clinical course illustrates the broad range of manifestations and the complexity of the diagnostic workup and (limited) therapeutic options for this condition.

## Case presentation

A 51-year-old woman who provided written informed consent for the publication of this case presented to the emergency department with progressive proximal muscle weakness involving both upper and lower extremities, which had begun approximately six weeks prior. She initially consulted her primary care physician, where moderate hypokalemia of 2.6 mmol/L (moderate hypokalemia ranges between 2.5 and 3.0 mmol/L) was detected. Concurrent diagnoses of type 2 diabetes mellitus and systemic arterial hypertension were made, and treatment was initiated with metformin 850 mg every eight hours, empagliflozin 25 mg once daily, and telmisartan 80 mg once daily. She denied the use of other medications or herbal supplements.

**Figure 1:**
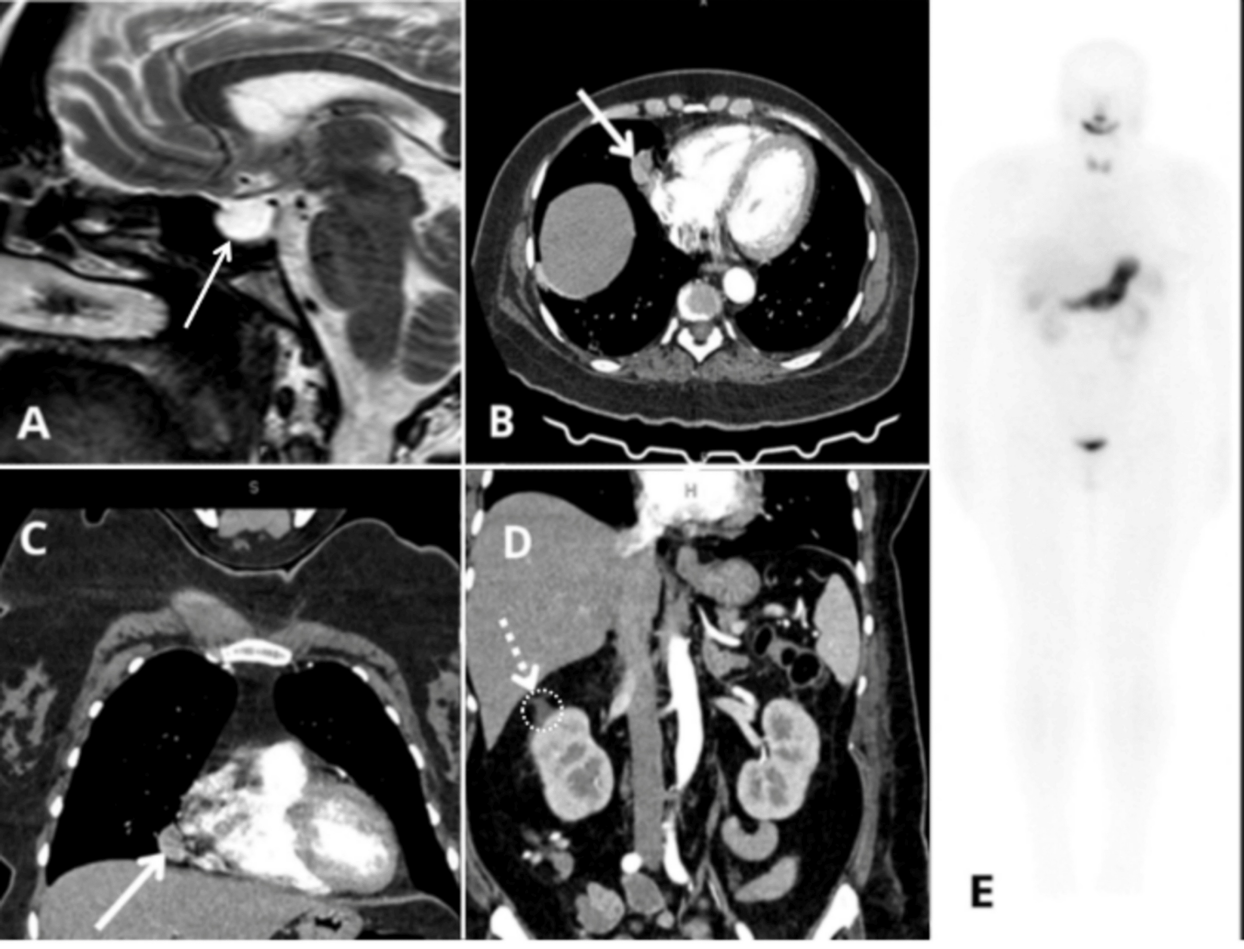
Serial CT and MRI imaging demonstrating multifocal lesions involving the pituitary gland, mediastinum, and adrenal gland (A) Sagittal T1-weighted brain MRI showing an expansive sellar arachnoidocele with downward displacement of the pituitary gland. (B, C) Contrast-enhanced chest CT in axial (B) and coronal (C) planes demonstrating an anterior mediastinal lymph node (arrows). (D) Contrast-enhanced abdominal CT revealing bilateral adrenal gland hyperplasia (arrow). (E) Somatostatin receptor scintigraphy with technetium-99m (^99m^Tc)-labeled octreotide showing no evidence of residual or metastatic disease.

Due to persistent muscle weakness, she sought further evaluation. On physical examination, her blood pressure was 150/90 mmHg. Notable findings included central obesity, facial plethora, hirsutism, and a dorsocervical fat pad. Neurological examination revealed proximal muscle strength of 3/5 in the lower limbs according to the Medical Research Council (MRC) scale. The initial laboratory analysis indicated metabolic alkalosis, severe hypokalemia, mild hypocalcemia, and abnormally increased urine chloride concentrations. The patient was admitted to the internal medicine department for additional assessment. Given the array of findings, such as arterial hypertension, chronic hypokalemia, proximal muscle weakness, and cushingoid characteristics, hypercortisolism was highly suspected. The endocrine assessment revealed significantly increased morning serum cortisol levels and excessive 24-hour urinary free cortisol excretion. Refractory hypokalemia persisted despite intravenous and oral potassium treatment. An overnight dexamethasone suppression test at a dosage of 1 mg revealed a lack of cortisol suppression, and subsequent 24-hour urinary cortisol measurements corroborated the presence of severe hypercortisolism. Furthermore, elevated ACTH levels corroborated the diagnosis of ACTH-dependent CS. The patient’s laboratory results are summarized in Table 1. Pituitary MRI showed no evidence of an adenoma but revealed an expansive sellar arachnoidocele with downward displacement of the pituitary gland due to diaphragmatic invagination. A contrast-enhanced thoracoabdominal CT scan identified a 24 × 14 mm anterior mediastinal lymph node with 60 Hounsfield units (HU) and no contrast enhancement. Bilateral adrenal gland hyperplasia was also noted, with measurements of 10.4 mm and enhancement from 47 HU (non-contrast) to 120 HU (venous phase). These findings are illustrated in Figure 1, including MRI, CT, and somatostatin receptor scintigraphy images.

**Table 1:** Initial laboratory results of the patient

Laboratory Test	Patient Value	Units	Reference Range
Serum potassium	2.2	mmol/L	3.5 – 5.0
Bicarbonate	35.7	mEq/L	22 – 29
Arterial pH	7.57	—	7.35 – 7.45
Ionized calcium	0.93	mmol/L	1.1 – 1.3
Urinary chloride	120	mmol/L	25 – 250
Morning serum cortisol	64	µg/dL	6 – 23
24-hour urinary free cortisol	>6,000	µg/day	20 – 90
Adrenocorticotropic hormone (ACTH)	798.1	pg/mL	< 46

**Figure 2:**
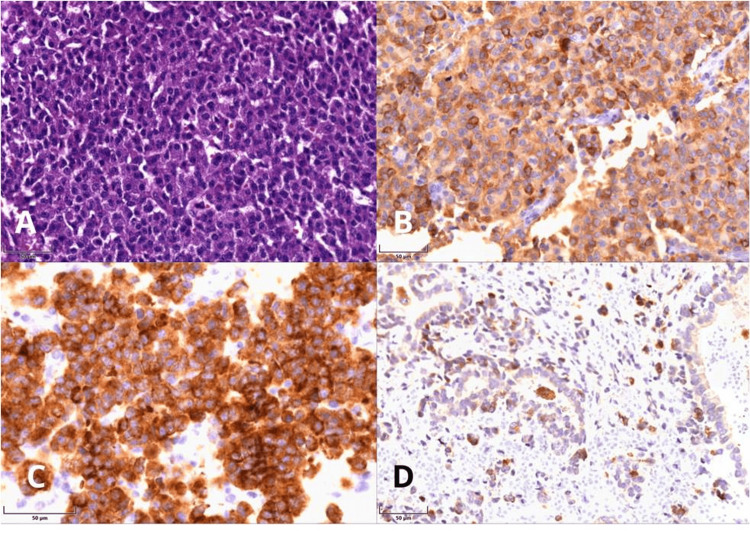
Mediastinal mass biopsy sheets A. Hematoxylin and eosin (H&E, 40×)–stained histological section showing a diffuse proliferation of small- to medium-sized cells with hyperchromatic nuclei and scant cytoplasm, replacing the parenchyma. B. Immunohistochemistry for chromogranin (40×) demonstrating diffuse cytoplasmic positivity in tumor cells. C. Immunohistochemistry for synaptophysin (60×) showing intense, diffuse granular cytoplasmic expression. D. Immunohistochemical staining for neuron-specific enolase (NSE) revealing focal cytoplasmic positivity in neoplastic cells. Final magnification: 40×; Scale bars: 50 µm.

Given the suspicion of ectopic ACTH secretion, the thoracic surgery team performed a complete video-assisted thoracoscopic surgery (VATS) resection of the mediastinal mass. Histopathologic analysis confirmed a well-differentiated grade 1 neuroendocrine tumor (pT1b, pNx, pMx) located in the lower lobe of the right lung. Immunohistochemistry demonstrated diffuse positivity for synaptophysin, chromogranin A, and neuron-specific enolase. Figure 2 summarizes the histopathological and immunohistochemical findings confirming neuroendocrine tumor differentiation.

The patient experienced prompt clinical and biochemical improvement following surgery. Within 24 hours, serum cortisol and ACTH levels declined to 11.1 µg/dL and 2.0 pg/mL, respectively, accompanied by normalization of serum potassium without further supplementation. She was discharged in stable condition under shared follow-up by the endocrinology and oncology services.

## Discussion

About 10% to 20% of individuals with CS have ECS, that is, an ACTH-producing tumor outside the pituitary gland. Patients with ECS frequently present with severe cases. A good outcome requires a methodical approach, tumor localization, control of cortisol excess, and prompt resection of the primary tumor [3].

Beyond the prompt localization of the tumor, recognizing the biochemical profile of ectopic ACTH secretion is critical, as these patients present with severe electrolyte disturbances. Such a finding may provide early clues to an ectopic source and guide the diagnostic approach.

In a case series of 58 patients with ECS, 24-hour urinary free cortisol levels greater than 4,000 µg were significantly associated with persistent and refractory hypokalemia, mirroring the profile seen in this case [5]. Elevated plasma ACTH, failure to suppress cortisol following the 1-mg dexamethasone test, and the absence of a pituitary lesion on MRI supported the diagnosis of an ectopic source. A contrast-enhanced thoracoabdominal CT scan identified a suspicious mediastinal mass, and histopathologic examination following VATS resection confirmed a well-differentiated neuroendocrine tumor.

Postoperative biochemical findings marked reductions in serum cortisol and ACTH, along with normalization of serum potassium without supplementation, indicated complete resection and biochemical resolution of ECS. Immunohistochemical analysis was consistent with a neuroendocrine neoplasm, showing strong cytoplasmic positivity for synaptophysin, chromogranin A, and neuron-specific enolase, further supporting the diagnosis of a hormonally active ACTH-secreting tumor. Postoperative somatostatin receptor scintigraphy using technetium-99m (^99m^Tc)-octreotide did not reveal any evidence of residual or metastatic disease. Although 68Ga-SSTR PET/CT imaging has demonstrated superior sensitivity in recent studies [4], ^99m^Tc-octreotide scans remain clinically valuable in postoperative staging and in resource-limited settings. The absence of residual disease on functional imaging, along with biochemical remission, confirms the therapeutic success of complete tumor resection.

This case underscores the importance of early recognition and systematic investigation of ACTH-dependent hypercortisolism. Rapid identification of an ectopic source and timely surgical intervention can lead to curative outcomes, even in severe biochemical presentations.

## Conclusions

ECS should be strongly considered in patients presenting with rapidly progressive hypercortisolism, refractory hypokalemia, and overt cushingoid features. A stepwise diagnostic approach, including biochemical confirmation, anatomical localization, and exclusion of pituitary disease facilitates timely identification of the underlying etiology. In cases such as this, prompt surgical resection of the ACTH-secreting tumor can result in definitive biochemical remission and resolution of clinical manifestations.
